# Inherited risk of dementia and the progression of cerebral small vessel disease and inflammatory markers in cognitively healthy midlife adults: the PREVENT-Dementia study

**DOI:** 10.1016/j.neurobiolaging.2020.10.029

**Published:** 2021-02

**Authors:** Audrey Low, Li Su, James D. Stefaniak, Elijah Mak, Maria-Eleni Dounavi, Graciela Muniz-Terrera, Karen Ritchie, Craig W. Ritchie, Hugh S. Markus, John T. O'Brien

**Affiliations:** a Department of Psychiatry, School of Clinical Medicine, University of Cambridge, Cambridge, UK; b Department of Clinical Neurosciences, University of Cambridge, Cambridge, UK; c Division of Neuroscience and Experimental Psychology, University of Manchester, Manchester, UK; d Centre for Dementia Prevention, University of Edinburgh, Edinburgh, UK; e INSERM, Montpellier, France

**Keywords:** Cerebral small vessel disease, White matter hyperintensities, Cerebral microbleeds, Inflammation, Alzheimer's disease, APOE4, Risk factors

## Abstract

Cerebral small vessel disease (SVD) and inflammation are increasingly recognized as key contributors to Alzheimer's disease (AD), although the timing, trajectory, and relation between them early in the disease process is unclear. Therefore, to investigate very early-stage changes, we compared 158 healthy midlife adults with and without inherited AD predisposition (APOE4 carriership (38% positive), parental family history (FH) of dementia (54% positive)) on markers of SVD (white matter hyperintensities (WMH), cerebral microbleeds), and inflammation (C-reactive protein (CRP), fibrinogen), cross-sectionally and longitudinally over two years. While WMH severity was comparable between groups at baseline, *longitudinal* progression of WMH was greater in at-risk groups (APOE4+ and FH+). Topographically, APOE4 was associated exclusively with *deep, but not periventricular,* WMH progression after adjusting for FH. Conversely, APOE4 carriers displayed *lower* CRP levels than noncarriers, but not fibrinogen. Furthermore, interaction analysis showed that FH moderated the effect of SVD and inflammation on reaction time, an early feature of SVD, but not episodic memory or executive function. Findings suggest that vascular and inflammatory changes could occur decades before dementia onset, and may be of relevance in predicting incipient clinical progression.

## Highlights

• Heritable risk factors related to SVD progression, despite lower levels of inflammation.
• SVD progression had more pronounced adverse effects on reaction time in at-risk individuals.
• Association between SVD and inflammation was stronger in those at-risk.

## 1 Introduction

Alzheimer's disease (AD) is a neurodegenerative disorder traditionally characterized by aberrant protein accumulation in the brain. In recent years, however, our understanding of AD etiology has expanded, implicating cerebral small vessel disease (SVD) and inflammation as key players in its pathogenesis (Bos et al., 2018; Heneka et al., 2015; Kinney et al., 2018; Wardlaw et al., 2013). However, the timing and contribution of these alterations early in the disease process remain unclear. This is an important gap to fill, as developing our understanding of vascular and inflammatory changes in the preclinical phase sheds light on the early trajectory and mechanistic pathways leading up to dementia, and thus facilitates early detection and disease management.

In AD, subclinical biological changes are detectable many years before observable symptom onset (Ritchie et al., 2016). Given the heritable nature of AD, one can identify at-risk individuals decades before disease onset would occur and thereby examine early alterations in asymptomatic individuals at higher risk of developing AD. Using this approach, we investigate longitudinal changes in SVD and inflammation in relation with established heritable risk factors, apolipoprotein ε4 (APOE4) (Strittmatter and Roses, 1996), and parental family history (FH) of dementia (Scarabino et al., 2016).

Radiological findings of SVD are common in the elderly population, and their presence predicts future dementia and risk of stroke (Debette et al., 2019). However, associations between inherited risk of dementia and SVD have been predominantly examined in cross-sectional studies, reporting largely discrepant findings depending on age cohorts. For instance, 2 studies of older adults, with mean ages of 64 and 74 years, respectively, reported significant associations between heritable risk factors and SVD (Stamm et al., 2019; Wolters et al., 2017). Yet, a study of younger adults—aged 52 years old on average—did not find evidence of such an association (Stefaniak et al., 2018). These contrasting reports suggest that the effect of inherited risk on vascular alterations may emerge only in older age and suggests a need to consider the longitudinal progression of neurovascular injury.

Inflammation is increasingly implicated in neurodegenerative diseases such as AD (Gabin et al., 2018; Gong et al., 2016; Heneka et al., 2015; Kinney et al., 2018; Tao et al., 2018). Under normal circumstances, inflammation is a protective biological response to infections and injuries. However, prolongation of an inflammatory response can have deleterious effects on surrounding tissue. Although the immune system is independent of the central nervous system, the two are engaged in constant bidirectional communication. In pathologic states, the central nervous system can be affected by systemic inflammation. Chronic systemic inflammation can compromise the integrity of the blood–brain barrier, thereby allowing the entry of toxins and pathogens into the brain, and activating glial cells (Bendorius et al., 2018; Kempuraj et al., 2017). Blood markers of inflammation have been linked to neurodegenerative conditions including AD (Gabin et al., 2018; Gong et al., 2016; Tao et al., 2018), cognitive decline (Noble et al., 2010; Watanabe et al., 2016), and SVD (Low et al., 2019). However, there has been a lack of research considering longitudinal changes in systemic inflammation in relation to heritable AD risk.

To address the existing gaps in literature, the core objective of our study was to investigate the influence of inherited dementia risk on the longitudinal progression of SVD and inflammation in cognitively healthy midlife adults. There is also significant merit in elucidating the pathologic contributions to early cognitive decline, as evidenced by recent findings that even subtle cognitive decline in preclinical AD has robust clinical utility in predicting disease progression and conversion (Papp et al., 2020). Therefore, we further examined the interaction between these pathologic markers and inherited risk factors on neuropsychological measures. In addition, we tested the association between SVD and inflammation and whether these relationships would be moderated by inherited risk. We hypothesized that SVD and inflammation would differ by APOE4 carriership and FH in terms of progression, but not cross-sectionally, and that these pathologies would be more detrimental to clinical measures in individuals with inherited predisposition to dementia, relative to those without.

## 2 Material and methods

### 2.1 Participants

The protocol of the PREVENT-Dementia study has been described in detail previously (Ritchie and Ritchie, 2012). Cognitively healthy, midlife (age 40–59 years) participants were recruited through multiple sources. Majority of participants with parental family history of dementia (FH+) were recruited from the dementia register database held at West London Mental Health National Health Service Trust, which holds information on patients with dementia and cognitive impairment who have consented to be approached for clinical research, and their careers (often offspring), or through memory clinic referrals—most FH+ participants therefore had parents with a confirmed diagnosis of dementia. On the other hand, majority of FH− participants were spouses/friends/other relatives of FH+ participants. Other participants were recruited via the Join Dementia Research website (https://www.joindementiaresearch.nihr.ac.uk/) or by registering their interest through the PREVENT-Dementia website (https://preventdementia.co.uk/) and public presentations and engagement sessions.

**Fig. 1:**
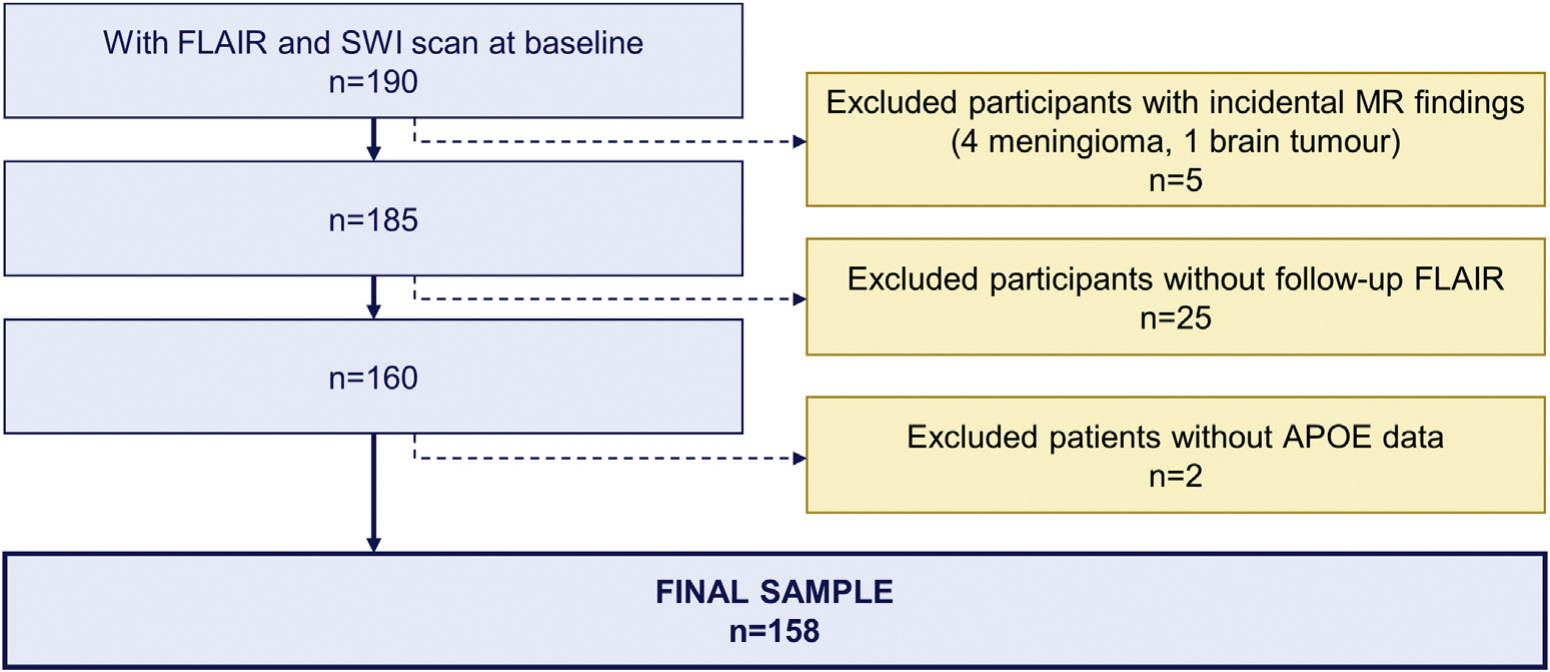
Participant selection flowchart.

This study included 158 participants who underwent clinical assessment and magnetic resonance imaging (MRI) at both baseline and two-year follow-up (mean = 2.03 years; range = 1.91–2.48 years) (Fig. 1). The research was approved by the London-Camberwell St Giles National Health Service Ethics Committee, and all subjects provided written informed consent. Family history of dementia was defined as positive (FH+) if participants reported at least one parent having clinically diagnosed dementia, while APOE4 status was regarded positive for ≥1 ε4 allele (APOE4+). Sixty were APOE4 carriers (38.0%), whereas 85 had a FH of dementia (53.8%), more often on the maternal (35.4%) rather than paternal side (13.3%), while 5.0% had both mothers and fathers with dementia. In terms of parental dementia subtype, majority of FH+ subjects reported Alzheimer's or mixed (Alzheimer's/vascular) dementia (n = 66, 76%), 13 reported vascular dementia (15%), and 8 were unsure or reported other dementia subtypes (9%). Four participants carrying both the APOE2 and APOE4 allele were analyzed as APOE4 carriers. Given that FH and APOE4 may have independent contributions to dementia risk (Donix et al., 2012), the two were analyzed as separate risk factors.

### 2.2 Imaging analysis

All participants underwent structural MRI at baseline and two-year follow-up. MRI scans were acquired on a 3T Siemens Verio. Three-dimensional T1-weighted MPRAGE parameters were: 160 slices, repetition time (TR) = 2300 ms, echo time (TE) = 2.98 ms, flip angle = 9°, voxel size = 1 × 1 × 1mm^3^. Fluid-attenuated inversion recovery (FLAIR) parameters were: 27 slices, TR = 9000 ms, TE = 94 ms, flip angle = 150°, voxel size = 0.43 × 0.43 × 4mm^3^. Susceptibility-weighted imaging (SWI) parameters were: 72 slices, TR = 28 ms, TE = 20 ms, flip angle = 15°, voxel size = 0.72 × 0.72 × 1.2 mm^3^.

### 2.3 Quantification of white matter hyperintensities

**Fig. 2:**
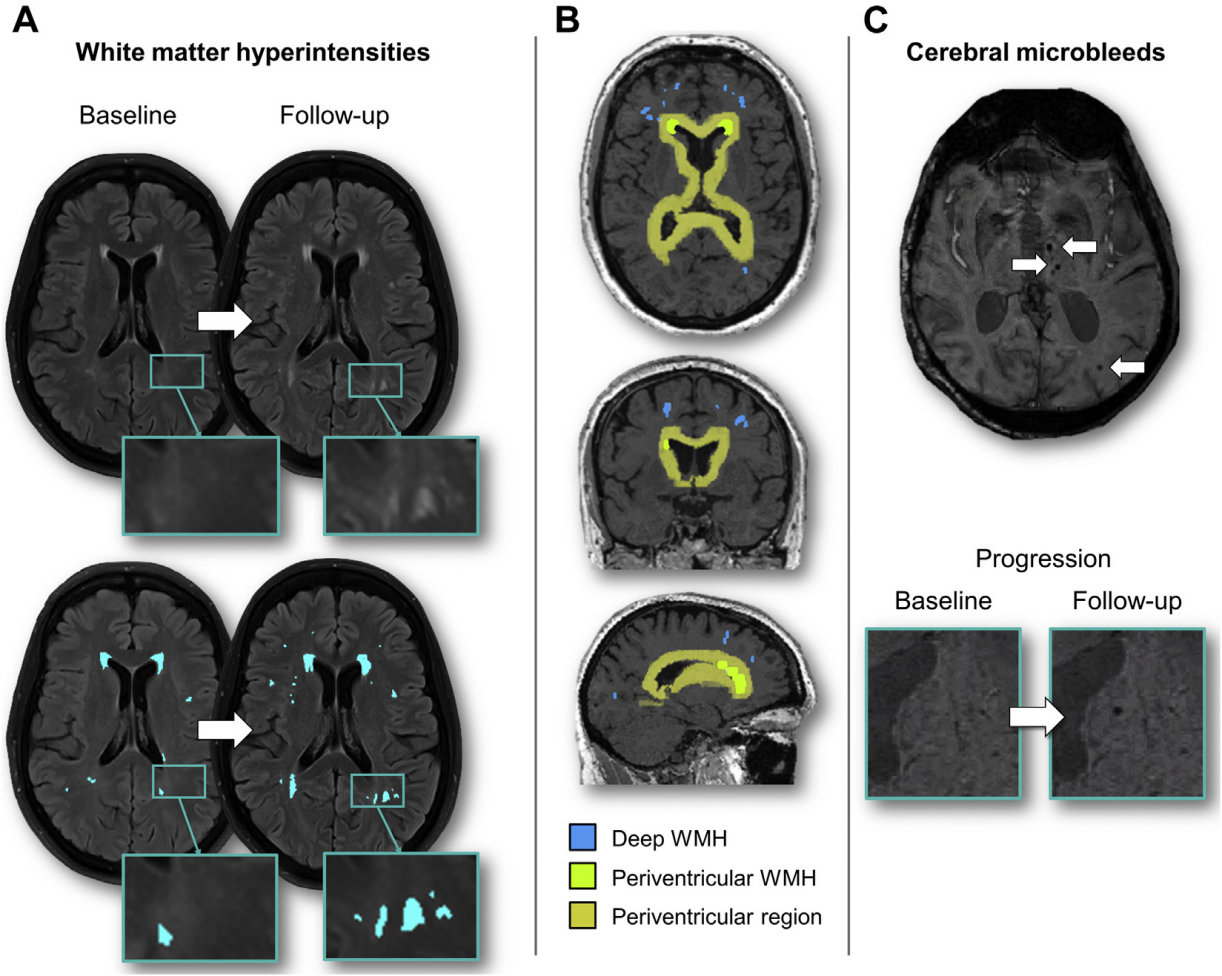
Radiological markers of cerebral small vessel disease (SVD). (A) White matter hyperintensities (WMH) were manually delineated on FLAIR MRI at baseline and two-year follow-up; (B) Classification of periventricular and deep WMH based on threshold distance from ventricles, derived from morphological ventricle dilation; (C) Cerebral microbleeds were identified on 3T SWI scans, and progression was defined as the occurrence of new microbleeds at follow-up, which were confirmed to be absent at baseline.

White matter hyperintensities (WMH) lesion maps were obtained using an automated script on the Statistical Parametric Mapping 8 (SPM8) suite (http://www.fil.ion.ucl.ac.uk/spm/) on FLAIR MRI; details on the procedures involved have been described previously (Firbank et al., 2003). Briefly, SPM8 was used to perform segmentation of T1-weighted images into gray matter (GM), white matter, and cerebrospinal fluid, based on prior probability maps. Using the GM and WM maps, a brain mask was created and used to perform removal of nonbrain matter from the FLAIR images. WMH segmentation was then conducted in FLAIR native space. Initial WMH maps were obtained using threshold-based segmentation at a threshold of 1.2 times the median pixel intensity, i.e., lesions with pixel intensity more than 1.2 times the median intensity of the whole brain were included in the WMH map. All lesion maps from both baseline and follow-up visits were reviewed by a single-experienced rater (A.L.) blinded to all clinical information, including FH, APOE4 status, and blood inflammatory levels (Fig. 2). Lesion maps obtained from the segmentation procedure were used as starting points for manual WMH delineation. Baseline and follow-up FLAIR images were evaluated side by side during delineation to ensure consistency. WMH volumes were normalized by total intracranial volume to account for individual differences in head size ((WMH/total intracranial volume) ∗ 100%) and transformed using cube root transformation due to skewness.

WMH were classified into periventricular and deep WMH based on threshold distance from ventricles. Binary masks of ventricles underwent 4 iterations of morphological dilation in MNI space. This dilated ventricle mask was then transformed to subject space to define the boundary between periventricular and deep WMH. Because of the transformation, this is a variable distance accounting for individual brain size and is approximately 10 mm, in line with recommendations (Griffanti et al., 2018).

### 2.4 Cerebral microbleed assessment

Cerebral microbleeds (CMB) were identified on 3T SWI following the Microbleed Anatomical Rating Scale (MARS) which quantifies CMB lesion count per location (Gregoire et al., 2009). In keeping with the MARS, lobar regions were defined according to Stark and Bradley (Stark and Bradley Jr, 1999), comprising cortical and subcortical regions, while deep regions included the basal ganglia, thalamus, internal capsule, external capsule, corpus callosum, and deep and periventricular WM (anatomical diagram available in Gregoire et al., 2009). Following the recommendations made by Greenberg et al. (2009), CMB were defined as areas of round/ovoid black signals, excluding tubular or linear structures (Greenberg et al., 2009) (Fig. 2). Suspected microbleeds were cross-validated on T1- and T2-weighted scans to exclude microbleed “mimics”. In instances of uncertainty, microbleeds were labeled as “possible microbleeds”—this includes situations whereby microbleeds cannot be distinguished from vascular flow voids. Such cases of “possible microbleeds” were excluded from analysis, and only “definite microbleeds” were analyzed. All CMB identified at follow-up were cross-checked against baseline scans to confirm their presence at baseline, and new CMB were flagged. Microbleed progression was defined as the presence of any new CMB at follow-up that were confirmed to be absent at baseline and was analyzed as a binary variable (new CMB vs. no new CMB). Ratings were performed blinded to all clinical information including FH, APOE4 carriership, and blood analysis.

### 2.5 Neuropsychological measures

Participants underwent neuropsychological assessment at baseline and two-year follow-up. As part of the COGNITO battery (Ritchie et al., 2014), participants were assessed on reaction time, episodic memory, and executive function. Reaction time was measured using a simple reaction time task administered through a touchscreen which records responses and response latencies, and mean reaction time across 12 trials was computed. Episodic memory was assessed using a delayed free recall task of 9 names, and executive function was evaluated using the Stroop test item of COGNITO.

### 2.6 Genotyping

TaqMan genotyping on QuantStudio12 K Flex was used to establish APOE variants. Genomic DNA was isolated from whole blood and genotyping was performed in 384 well plates, using the TaqMan polymerase chain reaction (PCR)-based method. The final volume PCR was 5 μL using 20 ng of genomic DNA, 2.5 μL of TaqMan Master Mix, and 0.125 μL of 40× Assay by Design Genotyping Assay Mix, or 0.25 μL of 20× Assay on Demand Genotyping Assay. The cycling parameters were 95° for 10 minutes, followed by 40 cycles of denaturation at 92° for 15 seconds and annealing/extension at 60° for one minute. PCR plates were then read on Thermo Fisher QuantStudio 12K Flex Real-Time System instrument with QuantStudio 12K Flex Software or TaqMan Genotyper Software v1.3.

### 2.7 Inflammatory markers

Systemic inflammation was quantified using serum measures of CRP and fibrinogen. Participants provided fasting blood samples on the morning of their first visit, the same day as clinical and cognitive assessments. Technicians were blinded to all clinical information. CRP concentration in serum was assessed using the Beckman Coulter AU System with a CRP Latex reagent. Following the latest recommendations of best practices, participants with CRP exceeding 10 mg/L (n = 6) were excluded from CRP analysis as these may be attributable to an acute immune response (Mac Giollabhui et al., 2020). Fibrinogen was measured using the Clauss method (Clauss, 1957) by determining the clotting time of diluted plasma after the addition of thrombin. CRP and fibrinogen data underwent cube root transformation due to right-tailed skewness.

### 2.8 Statistical analysis

Standard statistical techniques were used for descriptive analyses (Table 1). To test whether FH and APOE4 carriership differed in baseline levels of SVD (WMH and CMB) and inflammation (CRP and fibrinogen), we independently fitted regression models to each of these outcomes of interest (simple linear regression models fitted to WMH, CRP, and fibrinogen; logistic regression models fitted to binary CMB variable). In all models, we adjusted for sex (1 = male, 2 = female), age (centered at sample mean of 52.2 years), and years of education (centered at sample mean of 16.1 years) (corresponding results in Section 3.1). To assess whether FH and APOE4 carriership were associated with the trajectory of WMH, CRP, and fibrinogen, independent linear mixed effects models were fitted to each outcome of interest, allowing intercepts to vary between subjects and adjusted for the same covariates, while CMB progression was analyzed as a dichotomized variable (no new CMB vs. ≥1 new CMB) using logistic regression analysis (corresponding results in Section 3.2).

**Table 1:** Participant characteristics

Measures	Full sample158	Family history			APOE4		
		FH−	FH+	*p* value (FH)	APOE4−	APOE4+	*p* value (APOE4)
n	158	73	85		98	60	
Baseline demographics							
Sex (N, % females)^c^	109, 69.0%	49, 67.1%	60, 70.6%	0.639	68, 69.4%	41, 68.3%	0.889
Age in years^e^	52.2 (5.4)	51.1 (6.3)	53.1 (4.4)	0.113	52.7 (5.4)	51.3 (5.5)	0.083
Education in years^e^	16.1 (3.4)	16.4 (3.8)	15.9 (3.0)	0.206	16.0 (3.5)	16.3 (3.1)	0.581
Family history^c^	53.8%	-	100%	-	46.9%	65.0%	0.027^f^
APOE4^c^	38.0%	28.8%	45.9%	0.027^f^	-	100%	-
APOE2^c^	8.2%	8.2%	8.2%	0.997	9.2%	6.7%	0.576
Hypertension^c^	16.5%	11.0%	21.2%	0.084	16.3%	16.7%	0.955
Baseline							
WMH volume^a^^,^^e^	0.10 (0.15)	0.09 (0.13)	0.11 (0.17)	0.596	0.10 (0.17)	0.10 (0.13)	0.225
CMB (% present)^c^	21.5%	21.9%	21.2%	0.910	17.3%	28.3%	0.103
CRP (mg/L)^e^	2.7 (1.5)	2.7 (1.5)	2.8 (1.6)	0.586	2.9 (1.8)	2.4 (0.9)	0.030^f^
Fibrinogen	3.0 (0.7)	3.0 (0.7)	3.1 (0.7)	0.861	3.1 (0.7)	3.0 (0.6)	0.660
Reaction time (ms) b^,^^d^	341.1 (38.5)	334.9 (41.0)	346.5 (35.6)	0.063	338.7 (40.9)	345.0 (34.3)	0.304
Delayed recall	7.0 (1.5)	6.8 (1.6)	7.1 (1.4)	0.515	6.8 (1.6)	7.2 (1.5)	0.222
Stroop test	17.6 (3.1)	18.0 (3.0)	17.3 (3.2)	0.048^f^	17.7 (2.8)	17.4 (3.5)	0.549
Follow-up							
WMH volume^a^^,^^e^	0.12 (0.19)	0.10 (0.14)	0.14 (0.23)	0.182	0.12 (0.22)	0.12 (0.14)	0.035^f^
CMB (% present)^c^	29.9%	30.6%	29.4%	0.876	26.5%	35.6%	0.230
CRP (mg/L)^e^	2.8 (1.8)	2.8 (1.8)	2.9 (1.8)	0.261	3.0 (2.0)	2.6 (1.4)	0.423
Fibrinogen	3.0 (0.7)	2.9 (0.7)	3.1 (0.7)	0.027^f^	3.1 (0.8)	3.0 (0.7)	0.605
Reaction time (ms) b^,^^d^	343.5 (38.9)	339.2 (39.8)	347.0 (38.0)	0.262	344.3 (42.7)	342.2 (32.2)	0.732
Delayed recall	7.3 (1.5)	7.4 (1.6)	7.1 (1.4)	0.143	7.3 (1.6)	7.2 (1.4)	0.454
Stroop test	17.8 (3.1)	17.9 (3.5)	17.7 (2.9)	0.291	17.9 (3.5)	17.5 (2.5)	0.075

^a^ Values expressed as percentages (%), ^b,c^ Values as mean (standard deviation).

Key: CMB = cerebral microbleeds, CRP = C-reactive protein; FH = parental family history of dementia, WMH = white matter hyperintensities.

Missing data: CMB at follow-up (n = 1), baseline CRP (n = 1 missing; n = 5 outliers excluded), follow-up CRP (n = 3 missing; n = 2 outliers excluded), fibrinogen (n = 13), follow-up fibrinogen (n = 11), baseline reaction time (n = 2), follow-up reaction time (n = 5).

a Normalized volume adjusting for total intracranial volume ((WMH/TIV) ∗ 100%).

b Longer reaction time indicates poorer performance.

c Chi-square test of independence.

d *t*-test.

e Mann-Whitney *U* test.

f *p* < 0.05.

To test associations between SVD (WMH and CMB) and inflammation (CRP and fibrinogen) at baseline, regression models were fitted to WMH and CMB in separate models, and to examine whether these associations were moderated by group (FH, APOE4 carriership), we repeated the analysis with addition of *inflammation ∗ group* interaction terms (corresponding results in Section 3.3).

To assess the relationship between neuropsychological measures and biomarkers (WMH, CMB, CRP, and fibrinogen), linear regression models were independently fitted to neuropsychological measures, and moderation by heritable risk factors was tested by repeating the analysis with *biomarker ∗ group* interaction terms (corresponding results in Section 3.4). To examine whether the trajectory of WMH, CMB, CRP, and fibrinogen related to changes in reaction time, linear mixed effects models were fitted to neuropsychological measures; to test if these associations were moderated by FH or APOE4, *biomarker ∗ group ∗ interval* interactions were tested (corresponding results in Section 3.4).

All statistical analyses were conducted using R. Models were estimated under a missing at random missing data assumption using maximum likelihood estimation. The *lm* function was used to fit linear regression models, and *glm* was used for logistic regression models. From the *lme4* package (Bates et al., 2015), *lmer* was used to fit linear mixed effects models, and *glmer* was used for mixed effects logistic regression analysis. Statistical significance was set at *p* < 0.05.

## 3 Results

Sample characteristics are presented in Table 1. The sample was majority female (69%), had a mean age of 52.2 years (SD 5.4), and an average of 16.1 years of education (SD 3.4). Sex, age, and years of education did not differ by FH or APOE4 carriership.

### 3.1 Group differences at baseline

Using general linear models, APOE4 carriership was related to lower CRP levels and greater presence of CMB at baseline. On the other hand, WMH and fibrinogen did not differ by FH or APOE4 carriership (Table 2, Figure A.1).

**Table 2:** Baseline and longitudinal group differences by family history and APOE4

Measures	Standardized β	Family history		Standardized β	APOE4	
		t value	*p* value		t value	*p* value
Baseline						
WMH^a^	0.02	0.12	0.907	0.11	0.69	0.489
CMB^b^	−0.09	−0.44	0.660	0.41	2.03	0.042^d^
CRP^a^	−0.02	−0.15	0.885	−0.31	−2.01	0.047^d^
Fibrinogen^a^	−0.08	−0.48	0.632	−0.00	−0.03	0.980
Longitudinal change						
WMH^c^	0.05	2.24	0.026^d^	0.08	3.48	<0.001^e^
CMB^b^	−0.03	−0.09	0.931	0.33	1.12	0.262
CRP^c^	0.01	0.10	0.924	0.06	0.74	0.458
Fibrinogen^c^	0.07	1.02	0.311	−0.02	−0.22	0.828

Note: All models adjusted for sex, age, and years of education.

a General linear model.

b Logistic regression model.

c Linear mixed effects model.

d *p* < 0.05.

e *p* < 0.001.

### 3.2 Longitudinal group differences

**Fig. 3:**
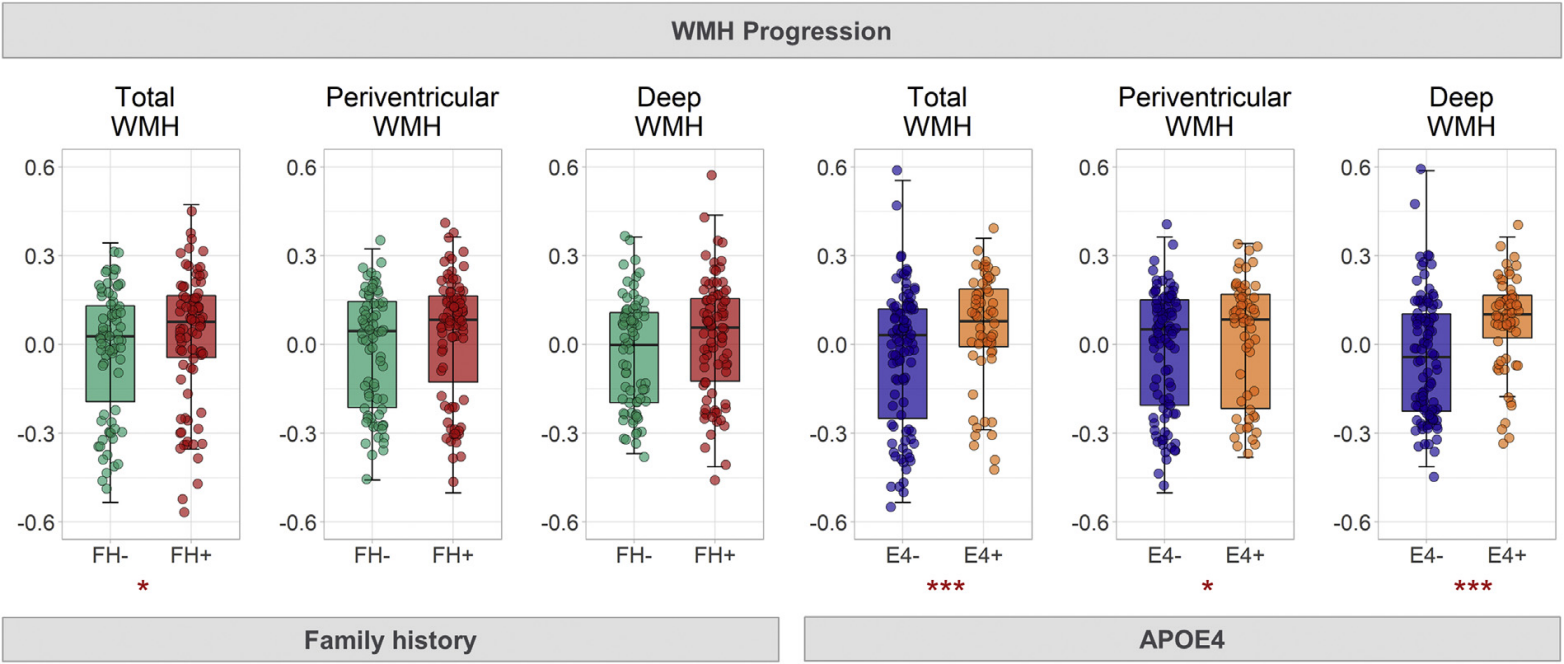
Boxplots depict greater WMH progression in participants with parental history of dementia (FH+) and APOE4 carriers (APOE4+). WMH volumes are residuals controlling for sex, age, and education. ∗*p* < 0.05, ∗∗*p* < 0.01, ∗∗∗*p* < 0.001.

Significant *FH ∗ interval* interaction in linear mixed effects modeling showed greater WMH progression in FH+ than FH− (Table 2, Fig. 3, Figure A.1). Linear mixed effects modeling of group differences (FH−, maternal FH+, and paternal FH+) further showed that maternal FH+ (β = 0.06, t = 2.32, *p* = 0.022), but not paternal FH+ (β = 0.05, t = 1.43, *p* = 0.155) related to greater WMH progression than FH−. Similarly, interaction analysis of *APOE4 ∗ interval* showed that APOE4 carriers had greater WMH progression than noncarriers (Table 2); this was observed for both periventricular (β = 0.06, t = 2.19, *p* = 0.030) and deep WMH (β = 0.10, t = 3.65, *p* < 0.001). Adjusting for FH, APOE4 remained significantly associated with deep (β = 0.10, t = 3.47, *p* < 0.001), but not periventricular WMH progression (β = 0.05, t = 1.92, *p* = 0.056). On the other hand, changes in CMB, CRP, and fibrinogen did not differ by FH or APOE4 carriership (Table 2).

### 3.3 Association between SVD and inflammation

In logistic regression analysis, *APOE4 ∗ CRP* interaction on CMB was significant at follow-up, whereby the relationship between CRP and CMB was more pronounced in APOE4 carriers than in noncarriers (β = 0.68, t = 2.76, *p* = 0.006); this applied to lobar CMB (β = 0.68, t = 2.68, *p* = 0.007), but not deep CMB (β < 0.01, t = 0.01, *p* = 0.992). Separate regression models confirmed that the positive associations between CRP and CMB were present in APOE4 carriers but not noncarriers.

### 3.4 Reaction time

Interaction analysis in general linear models showed that FH moderated the association between WMH and longer reaction time, in that the relationship was more pronounced in FH+ rather than FH− (total WMH: β = 0.52, t = 3.11, *p* = 0.002; periventricular WMH: β = 0.58, t = 3.49, *p* < 0.001; deep WMH: β = 0.37, t = 2.25, *p* = 0.026). In terms of inflammation, significant interaction of CRP and FH on reaction time showed that the relationship between higher CRP levels and longer reaction time was stronger in FH− than FH+ (β = −0.38, t = −2.41, *p* = 0.017).

**Fig. 4:**
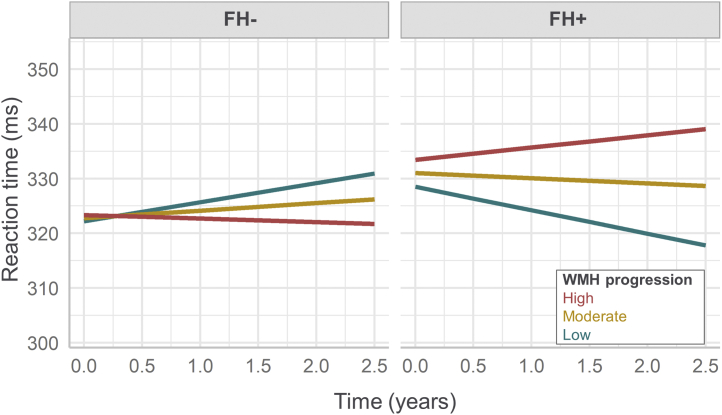
Plot of estimated marginal means of reaction time depicts a significant three-way interaction of time (scan interval), family history, and total WMH progression on reaction time from linear mixed effects regression model. Note: Increasing reaction time equates to poorer performance. *High* and *low* WMH progression was defined as +/−1 SD from mean (*moderate* WMH progression). Linear mixed model adjusted for sex, age, and education.

Longitudinally, significant *FH ∗ WMH ∗ interval* interaction in mixed effects analysis demonstrated that the association between WMH progression and increase in reaction time was stronger in FH+ than FH− (β = 0.15, t = 2.09, *p* = 0.038) (Fig. 4). This effect was observed in deep (β = 0.19, t = 2.77, *p* = 0.006) but not periventricular WMH (β = 0.08, t = 1.13, *p* = 0.262). Independent models fitted in the FH+ and FH− groups separately confirmed that the positive association was significant in FH+ but not the FH− group. Similarly, FH moderated the association between CMB progression and longitudinal change in reaction time, in that the relationship was stronger in FH+ than FH− (β = 0.15, t = 2.12, *p* = 0.036). These interaction effects were not observed in relation to episodic memory or executive function.

## 4 Discussion

We examined the effect of inherited predisposition to dementia (parental history of dementia and/or the APOE4 allele) on SVD and systemic inflammation in healthy midlife adults at baseline and longitudinally. Inherited risk was associated with lower levels of CRP, higher incidence of CMB, and greater increase in WMH over two years. Furthermore, the detrimental effect of SVD on reaction time was more pronounced in those with FH relative to those without, cross-sectionally and longitudinally. Conversely, the relationship between elevated CRP levels on reaction time was stronger in those without FH.

Increased WMH can be detected years before estimated symptom onset in dominantly inherited AD, which suggests that WMH may be an early event in AD pathogenesis (Lee et al., 2016). In this study, although WMH severity did not differ by FH or APOE4 carriership at baseline, these risk factors were associated with greater *longitudinal* progression of WMH over a two-year period. Adjusting for FH, APOE4 was associated exclusively with *deep* (but not periventricular) WMH progression. This may be linked to etiological differences between periventricular and deep WMH. Some studies have found that only periventricular WMH is implicated in increased risk of AD (Kim et al., 2015; Van Straaten et al., 2008), whereas both periventricular and deep WMH are associated with subcortical vascular dementia (Kim et al., 2015). Therefore, the exclusive coupling of APOE4 and deep WMH could be taken to imply that APOE4 predisposes one to dementia via vascular pathways. This is aligned with earlier studies showing that associations between AD severity and WMH progression were diminished after adjusting for APOE4 status, suggesting that WMH accumulation may be driven by APOE4 rather than AD diagnosis (Sudre et al., 2017). On the other hand, the heightened predisposition to dementia associated with parental history extends beyond genetics, and may also be driven by concomitant nongenetic factors such as shared environment, lifestyle, and socioeconomic status (Scarabino et al., 2016). In addition, we observed that greater WMH progression was observed in those with maternal but not paternal family history of dementia, although unequal sample sizes warrant replication of this finding in a more balanced sample.

In our sample of healthy midlife adults, APOE4 carriers displayed *lower* levels of CRP, a sensitive marker of systemic inflammation (Pepys and Hirschfield, 2003). Given that SVD and CRP are known to be *positively* associated (Low et al., 2019), it is counterintuitive that a risk factor (in this case, APOE4) would be associated positively with one pathology but negatively with another. Although somewhat surprising at first glance, these results are corroborated by earlier studies similarly reporting *lower* levels of CRP in APOE4 carriers (Chasman et al., 2006; Grönroos et al., 2008; Haan et al., 2008; Hubacek et al., 2010; Mänttäri et al., 2001; März et al., 2004; Ukkola et al., 2009). The juxtaposition of these contradictory findings suggests that APOE4 and CRP could be operating along distinct mechanistic pathways in relation to SVD, and may offer novel insight into the causal pathways of APOE4 as a risk factor in neurodegeneration. Despite being well documented in the literature, the biological mechanisms responsible for the negative association remain to be elucidated, although some attribute it to the downregulation of the mevalonate pathway in APOE4 carriers (März et al., 2004). CRP and fibrinogen levels did not differ by family history—this is consistent with the only other study to our knowledge examining CRP in relation to parental history of dementia, which also reported nonsignificant findings (Van Exel et al., 2009).

Inherited dementia risk had a dual effect on disease progression, in that predisposed individuals not only experienced greater progression of SVD, but also more pronounced slowing of reaction time in relation to SVD progression. In other words, the same degree of pathologic progression was related to greater slowing of reaction time in a person with inherited risk, than in a person without. This suggests that at-risk individuals could have a heightened susceptibility to vascular alterations. An alternative explanation is that SVD progression linked to heritable risk factors may be accompanied by other risk-related pathologies contributing to the cascade of clinical deficits (Shi et al., 2017). In terms of inflammation, elevated CRP levels were associated with slower reaction times, particularly in those *without* parental history of dementia. The absence of this association in those with family history suggests that poorer clinical features in this group may be driven instead by incipient disease. Notably, these findings were observed only in relation to reaction time but not other neuropsychological measures, i.e., episodic memory and executive function. This may be due to reaction time being a particularly early clinical feature in SVD (Jouvent et al., 2015; Richards et al., 2019), compared with the cognitive measures of memory and executive function which may be affected in later disease stages.

Positive associations between SVD and CRP were present in individuals with inherited risk but not in those without. This is in line with existing literature demonstrating the moderating effect of APOE4 on the relationship between inflammation and SVD (Low et al., 2019; Romero et al., 2012; Walker et al., 2017). Specifically, APOE4 has been shown to moderate associations between CRP and WMH (Walker et al., 2017) and between lipoprotein phospholipase A2 and CMB (Romero et al., 2012). This may be attributed to the increased propensity of APOE4 to promote inflammation and the tendency for APOE4 carriers to produce stronger neuroinflammatory responses to peripheral systemic inflammation (Cash et al., 2012; Lynch et al., 2003; Ophir et al., 2005). However, due to the inability to ascribe directionality, alternative interpretations should also be considered, whereby CRP acts as a moderator, i.e., APOE4 relates to greater SVD severity, but only in individuals with high CRP levels. To that end, the finding that high CRP levels amplified the detrimental effect of APOE4 on SVD suggests differential vulnerability, whereby individuals with heritable risk are more susceptible to vascular risk factors and consequent parenchymal injury. This corresponds with existing evidence that APOE4 has a lower antioxidant capacity than other isoforms and is less effective in protecting against oxidative stress, both of which could exacerbate the deleterious effects of risk factors such as smoking and low-grade inflammation (Dose et al., 2016; Jofre-Monseny et al., 2008; Miyata and Smith, 1996). Although we are unable to conclude the genetic overlap between SVD and AD in this present study, past research suggests that AD shares some genetic overlap specifically with SVD-related stroke but not other stroke subtypes (Traylor et al., 2016). Nevertheless, the relationship between the genetics of SVD and AD remains poorly understood at present and warrants further investigation.

Biases in this study could have arisen from SVD ratings not being blinded to time point. Baseline and follow-up WMH lesion maps were compared side-by-side to improve consistency of WMH delineation, while SWI scans from both time points were compared with confirm that “new” CMB in follow-up scans were not present at baseline. While intended to improve accuracy, the lack of blinding to time point may have introduced implicit bias toward the detection of greater SVD burden at follow-up. That being said, such systemic biases would apply to the whole sample and should not have affected the results on the group differences observed. Furthermore, the degree of WMH and CMB progression observed falls within the range of annual progression in previous reports (Alber et al., 2019; Harper et al., 2018).

CMB incidence was higher than earlier reports in healthy populations, likely due to (1) more sensitive detection of CMB afforded by thin slice 3T SWI, which detects up to 3 times the number of CMB compared with conventional 1.5 T GRE (Nandigam et al., 2009), and (2) oversampling of participants with a family history of dementia and, by extension, APOE4 carriership, a well established risk factor for CMB in older adults (Poels et al., 2010). Majority of CMB positive cases had a single microbleed, while only a handful had more than one. Because of the dichotomization of CMB burden (present/absent), our ability to make inferences based on the degree of CMB severity was limited.

A key strength of our study was its longitudinal design, which enabled the investigation of pathologic progression across time. Furthermore, we focused on a relatively young subclinical sample of midlife adults, with and without heritable risk factors, which allowed us to identify early biomarker changes in individuals at risk of developing dementia. Several limitations warrant the replication of results, including the relatively modest sample size, lack of correction for multiple comparisons, missing values which may introduce bias in the estimation of parameters, and FLAIR slice thickness of 4 mm which may represent a limitation on WMH quantification. In addition, inflammation was measured using peripheral blood markers, which may not be reflective of inflammation in the central nervous system. CRP levels are known to vary widely over a short timescale, which could perhaps explain the presence of associations between CRP and APOE4 at baseline but not at follow-up. Furthermore, type of parental dementia was not limited to AD but included mixed and vascular dementia, in which inherited risk may propagate primarily vascular but not AD pathology, while diagnostic imprecision of parental dementia type is another limitation. While FH+ participants were largely recruited from dementia registries or through memory clinic referrals, FH information of participants recruited from other sources relied on self-report and was thus more susceptible to misreporting. Furthermore, those recruited from other sources, including FH− participants, were observed to be less motivated and less likely to return for follow-up assessments, resulting in greater dropout rates within this subset. Finally, as the sample was predominantly white (90%) (Ritchie et al., 2017), findings may not be applicable across other ethnicities. Future studies with larger, multiethnic cohorts and longer follow-up periods will be crucial to understand the interaction between these pathologic processes across the adult lifespan.

## 5 Conclusions

Our data demonstrate that cerebrovascular alterations can be detected decades before dementia onset would occur in those at risk. Individuals with parental history of dementia and/or APOE4 carriership experienced greater progression of cerebrovascular disease, despite showing lower levels of inflammation. Progression of cerebrovascular pathology also had more pronounced adverse effects on reaction time in at-risk individuals. Taken together, our findings suggest that inherited risk factors may accelerate the progression of cerebrovascular damage as a function of heightened vulnerability to vascular risk and low-grade inflammation, as opposed to direct causative mechanisms. Clinically, our findings signal the relevance of SVD and inflammation in relation to early clinical features and disease trajectory.

## Disclosure statement

Authors declare no conflicts related to this study. Unrelated to this work, JOB has received honoraria for work as DSMB chair or member for TauRx, Axon, Eisai; has acted as a consultant for Lilly; and has received honorarium for talks from GE Healthcare and research support from Alliance Medical.
