# Supplementary material for: Inherited risk of dementia and the progression of cerebral small vessel disease and inflammatory markers in cognitively healthy midlife adults: the PREVENT-Dementia study
Source: Neurobiol Aging. 2021 Feb;98:124–33. doi: 10.1016/j.neurobiolaging.2020.10.029 (PMC7895800; doi:10.1016/j.neurobiolaging.2020.10.029)
Supplement: Supplementary Material [file mmc1.docx]

**SUPPLEMENTARY MATERIAL**

**
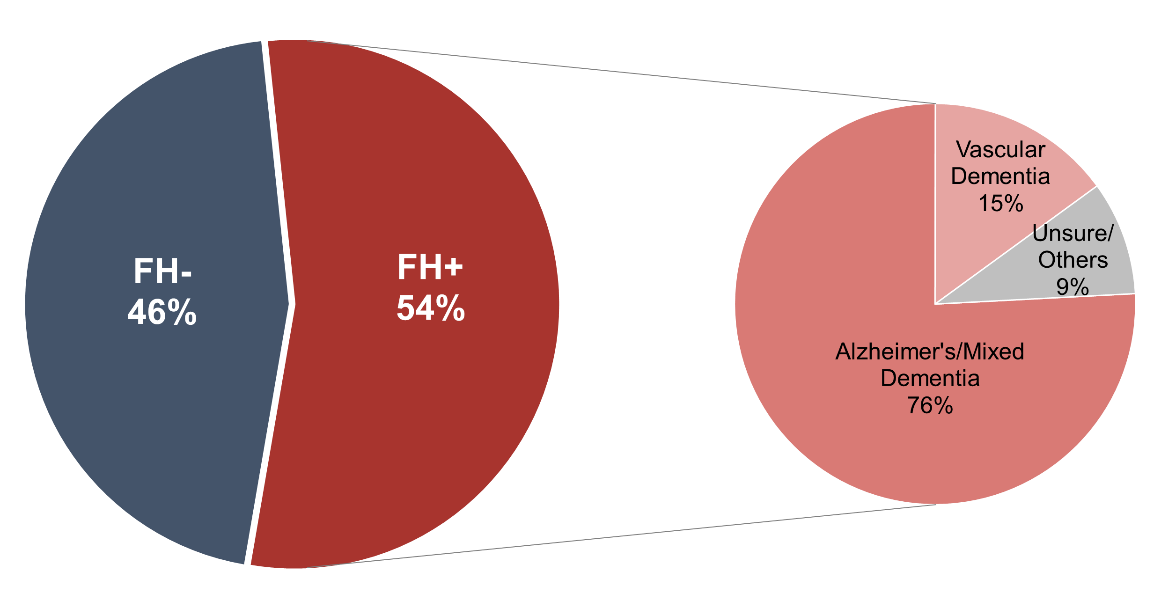
**

**Figure A.1. Composition of family history dementia subtypes.**


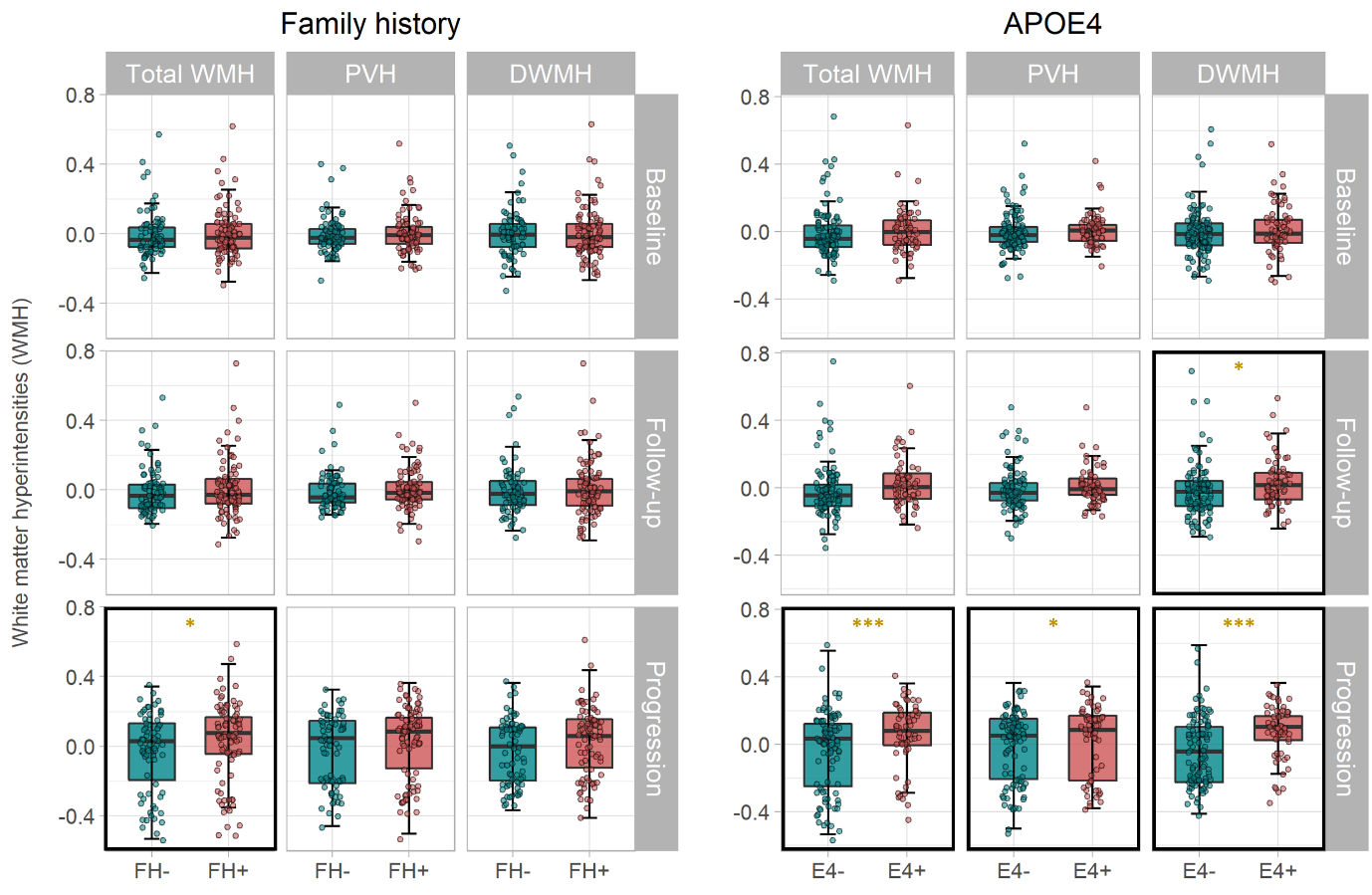


**Figure A.2. Group and regional differences in white matter hyperintensity volume at baseline, follow-up, and longitudinal change.** Significant group differences are highlighted in black boxes. * p<.05, **p<.01, ***p<.001.


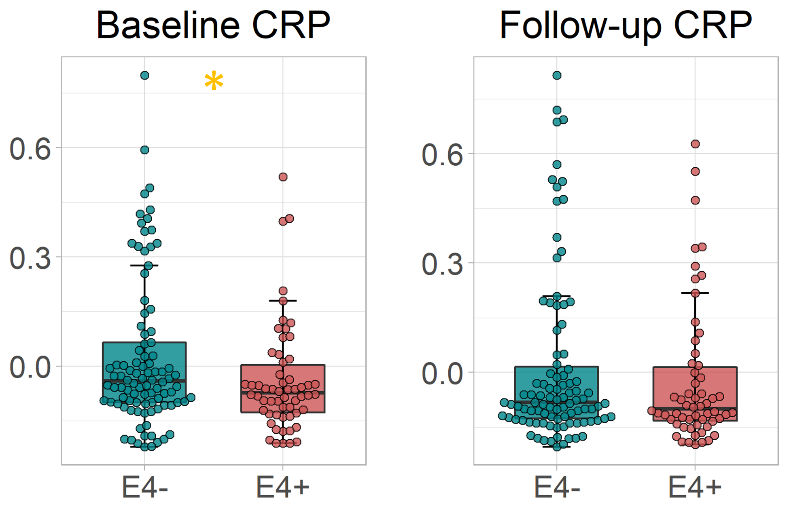


**Figure A.3. CRP levels were lower in APOE4 carriers, compared to non-carriers.** Values plotted are residuals adjusting for sex, age, education.

**
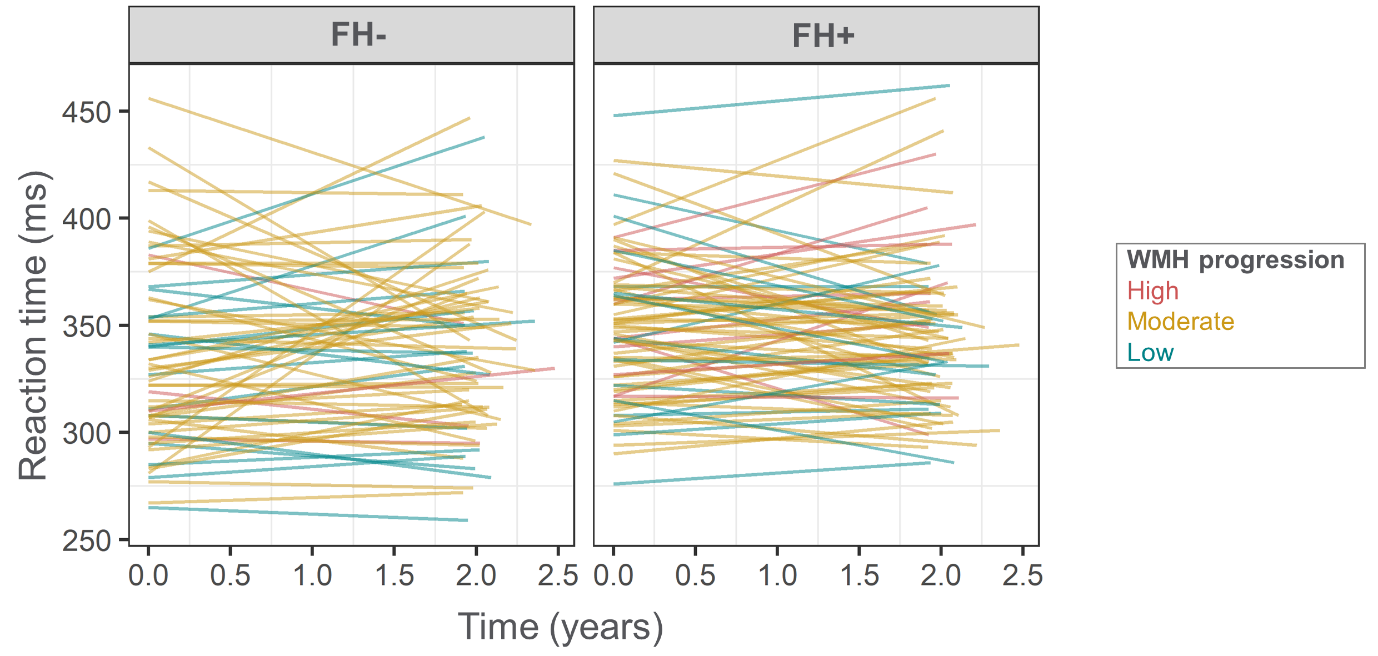
**

**Figure A.4. Subject-level trajectories of longitudinal change in reaction time**
